# Lymphocytic thrombophilic arteritis, an infrequent cause of livedo reticularis – case report^☆^

**DOI:** 10.1016/j.abd.2025.501148

**Published:** 2025-07-14

**Authors:** Dominga Peirano, Maria Luz Hubner-Garretón, Martha Fernandez, Andrés Figueroa

**Affiliations:** a Department of Dermatology, Clínica Dávila Recoleta, Santiago, Chile; b Department of Dermatology, Clínica Universidad de los Andes, Santiago, Chile; c Department of General Medicine, Clínica Redsalud Providencia, Santiago, Chile; d Department of Pathology, Clínica Dávila Recoleta, Santiago, Chile

*Dear Editor,*

Lymphocytic thrombophilic arteritis (LTA), also known as macular lymphocytic arteritis (MTA), is a small-to-medium vessel arteritis. LTA shares many similarities with cutaneous polyarteritis nodosa (cPAN) i.e livedo reticularis but does not exhibit systemic features like c-PAN.^1^ The objective of this report is to present the first documented case of LTA in a Chilean patient.

A 27-year-old female consulted in dermatology service asking for a second opinion in the context of a prior history and diagnosis of cutaneous leukocytoclastic vasculitis (CLV) of two years of duration, which had been treated with corticosteroids without success.

She reported an increase in lesions, accompanied by fatigue and arthralgias in the lower extremities. She denies having arthritis, oral ulcers, photosensitivity, symptoms of sicca syndrome, or neuropathy. Physical examination revealed non-palpable confluent hyperpigmented macules with a livedoid distribution located on the legs, arms, and trunk, with mild infiltration (Fig. 1). Only two scars were observed in the internal malleolar area (attributed by the patient to footwear), with no ulcers or cutaneous pain. There were no abnormalities detected in the lymph nodes, thyroid, breasts, chest, abdomen, or joints.

**Fig. 1:**
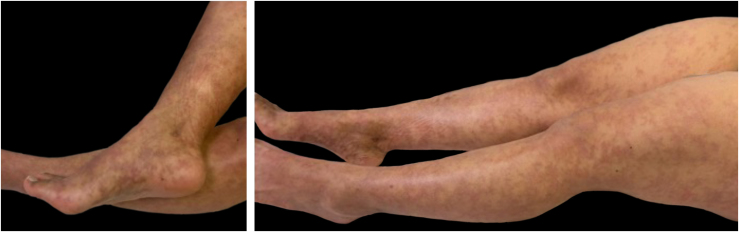
Non-palpable confluent hyperpigmented macules with a livedoid distribution are observed on the legs and feet.

Laboratory tests showed a slight elevation of the erythrocyte sedimentation rate (ESR) and a myositis panel strongly positive for threonyl-tRNA synthetase (PL-7). Antinuclear antibody (ANA) was positive at 1:80 with a fine granular pattern (AC-4), while other blood tests were negative (extractable nuclear antigen [ENA], rheumatoid factor [RF], anti-cyclic citrullinated peptide [anti-CCP], anti-double stranded DNA [anti-DNA], perinuclear anti-neutrophil cytoplasmic antibodies [ANCA-p], and cytoplasmic ANCA [ANCA-c]) or within normal levels (complement C3, C4, renal, and liver function tests). There was no evidence of anemia, leukocytosis, or thrombocytopenia. A chest Computed Tomography (CT) scan revealed no pathological findings.

A biopsy performed two years ago described: “Small vessels with abundant fibrin in the walls and scant inflammatory cells, primarily neutrophils, with minimal leukocytoclasis. Deep vessels showed no significant morphological alterations” which was consistent with CLV.

Given the diagnosis of macular cutaneous vasculitis without systemic features and the strong positivity for PL-7, further tests (ANA, myositis panel, Creatine Kinase [CK], Hepatitis B Virus [HBV], Hepatitis C Virus [HCV], Human Immunodeficiency Virus [HIV]) were conducted, confirming elevated PL-7 again with all other results negative. A new punch biopsy was requested, and initial treatment with hydroxychloroquine (Plaquinol) was initiated.

The patient returned with the new biopsy results (Fig. 2). The pathology service suggested considering lymphocytic macular arteritis, vasculitis associated with connective tissue disease (including lupus, rheumatoid arthritis, morphea/scleroderma, and Sjögren's syndrome), Behçet's disease, and viral infections based on histopathological patterns. Clinical suspicion leaned towards lymphocytic thrombophilic arteritis.

**Fig. 2:**
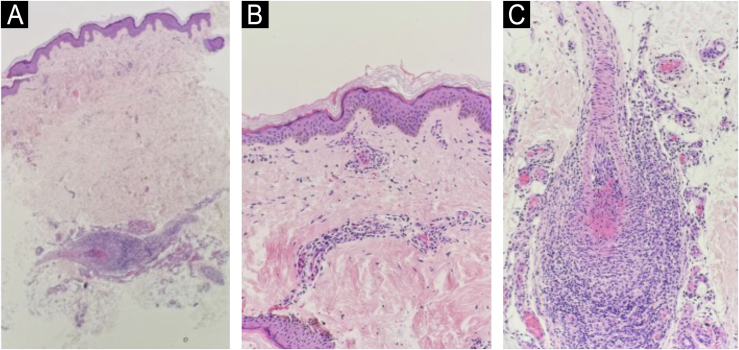
(A) Low magnification view (×10) showing orthokeratosis and a preserved epidermis. There is erythrocyte extravasation with scant lymphocytic infiltrate around the walls of superficial and deep dermal postcapillary venules. A medium-caliber vessel exhibits fibrin deposition within the intima and subtotal to total luminal occlusion. (B) Medium magnification view (×40) highlighting a closer look at the epidermis and the underlying dermis. Erythrocyte extravasation is evident, and a mild lymphocytic infiltrate is seen in the superficial dermis, with preserved tissue architecture and no signs of granulomas, leukocytoclasis, or interstitial dermal mucinosis. (C) High magnification view (×100) focusing on a dense mixed infiltrate predominantly composed of mononuclear cells, including lymphocytes, plasma cells, and histiocytes, along with a few polymorphonuclear cells. There is no perivascular tissue involvement, nor any granulomas, leukocytoclasis, or thickened basement membrane, consistent with deep dermal small and medium vessel vasculitis without leukocytoclasia.

The patient developed frank Raynaud's phenomenon (previously non-existent), hand arthralgia, and alopecia, without sicca symptoms, reflux, or dysphagia. Treatment was initiated with acetylsalicylic acid, oral nifedipine, and topical nifedipine and arnica with good response.

In conclusion, LTA is an infrequent arteritis and is probably underreported. To the best of our knowledge, this is the second case report in Latin America.

The clinical presentation of LTA is characterized by the appearance of hyperpigmented macules primarily on the lower limbs, and less commonly on the upper limbs, particularly in women over 40 years old. Additionally, livedo racemosa/reticularis or nodules may be observed, underscoring the clinical resemblance to c-PAN.^2^ Both conditions often manifest in a similar distribution and morphology, making the differentiation challenging based solely on clinical features.^3^

Histologically, LTA is distinguished by lymphocytic infiltration and thrombophilic features in the dermal and subcutaneous vessels. Key characteristics include luminal fibrin deposition and a hyalinized fibrin ring within the vessel lumen, both of which are markers of a thrombophilic state. The intense lymphocytic infiltration surrounding the affected vessels in LTA also resembles cPAN in its chronic stages. However, while LTA primarily shows lymphocytic involvement, cPAN may transition from a neutrophilic to a lymphocytic infiltrate as the disease progresses through acute to reparative stages. The shared features of luminal fibrin deposition and lymphocytic infiltration create histopathologic overlaps with cPAN, yet LTA generally lacks the systemic manifestations commonly associated with cPAN.^3^, ^4^

Treatment of LTA remains challenging as there are few reported cases, leading to a lack of consensus or studies supporting a definitive treatment approach. Further research is needed to establish the most effective treatment strategies for this condition.

In summary, LTA and cPAN share clinical and histopathologic similarities, such as the presence of livedo-like lesions and lymphocytic vascular infiltration. However, LTA is typically confined to the skin with a more indolent course, while cPAN carries the potential for systemic involvement, painful nodules, and other inflammatory features not usually present in LTA.^3^

## Financial support

None declared.

## Authors’ contributions

Dominga Peirano: Study concept and design; Data collection, or analysis and interpretation of data; Writing of the manuscript or critical review of important intellectual content; Final approval of the final version of the manuscript.

Maria Hubner-Garretón: Study concept and design; data collection, or analysis and interpretation of data; Writing of the manuscript or critical review of important intellectual content; Final approval of the final version of the manuscript.

Martha Fernandez: Study concept and design; Intellectual participation in the propaedeutic and/or therapeutic conduct of the studied cases; Critical review of the literature; Final approval of the final version of the manuscript.

Andrés Figueroa: Study concept and design; intellectual participation in the propaedeutic and/or therapeutic conduct of the studied cases; Critical review of the literature; Final approval of the final version of the manuscript.

## Conflicts of interest

None declared.
